# Myricitrin Alleviates Hypercholesterolemia and Non-Alcoholic Fatty Liver Disease in High Cholesterol Diet-Fed Mice

**DOI:** 10.3390/nu17030415

**Published:** 2025-01-23

**Authors:** Young-Je Kim, Sojeong Park, HwiCheol Kim, Sang Ryong Kim, Un Ju Jung

**Affiliations:** 1 Department of Food Science and Nutrition, Kyungpook National University, Daegu 41566, Republic of Korea; breezy750@naver.com; 2 Department of Food Science and Nutrition, Pukyong National University, 45 Yongso-ro, Nam-gu, Busan 48513, Republic of Korea; impark-sj@naver.com (S.P.); k423897@naver.com (H.K.); 3 School of Life Sciences, Kyungpook National University, Daegu 41566, Republic of Korea; srk75@knu.ac.kr

**Keywords:** myricitrin, high-cholesterol diet, hypercholesterolemia, non-alcoholic fatty liver disease

## Abstract

Background/Objectives: This research investigated the effects of myricitrin on hypercholesterolemia and non-alcoholic fatty liver disease (NAFLD) in mice given a high-cholesterol diet (HCD). Methods: C57BL/6J mice were maintained for 20 weeks on an HCD with or without myricitrin. Results: Myricitrin had no impact on the food consumption, body weight, or plasma triglyceride concentrations. However, myricitrin-supplemented mice had lower plasma total cholesterol (TC) concentrations and LDL + VLDL-cholesterol/TC proportion, and higher HDL-cholesterol/TC proportion than control mice, which resulted in a markedly decreased atherogenic index. Moreover, the levels of plasma C-reactive protein, oxidized LDL, lipoprotein(a), and plasminogen activator inhibitor-1, which are indicators for cardiovascular disease (CVD), were reduced, while levels of plasma paraoxonase, a cardioprotective enzyme, were greater in myricitrin-supplemented mice than in control mice. Myricitrin also meaningfully reduced liver weight and hepatic cholesterol content, and slightly alleviated fatty liver and fibrosis caused by an HCD. The plasma and hepatic cholesterol-lowering effects of myricitrin were partly associated with decreased activities of hepatic 3-hydroxy-3-methylglutaryl-CoA reductase and acyl-CoA:cholesterol acyltransferase, which are involved in cholesterol synthesis and esterification, respectively, as well as mRNA expression. Myricitrin also altered other hepatic genes implicated in cholesterol homeostasis, including the downregulation of SREBP2 and ABCA1 mRNA expression and the upregulation of LDLR mRNA expression. Moreover, myricitrin decreased TBARS levels in the liver and erythrocytes by activating antioxidant enzymes (SOD and catalase). Conclusions: These results indicate that dietary myricitrin may offer therapeutic benefits for HCD-caused hypercholesterolemia and NAFLD, and may help reduce CVD risk.

## 1. Introduction

Dietary cholesterol is a critical factor that influences the occurrence and worsening of dyslipidemia, non-alcoholic fatty liver disease (NAFLD), and cardiovascular disease (CVD). Although the effects of ingested cholesterol on blood lipid concentrations and subsequent disease risk can vary across different species and individuals, evidence suggests that excessive dietary cholesterol intake can induce hypercholesterolemia, a key driver of CVD [1,2,3]. Moreover, high cholesterol intake can contribute to abnormal hepatic lipid accumulation and NAFLD progression. Simple steatosis is further aggravated by hepatocyte injury, inflammation, and fibrosis [4]. NAFLD independently contributes to negative cardiovascular outcomes and death [5,6]. Given the significant role of dietary cholesterol in these metabolic disorders, exploring dietary interventions that can mitigate its harmful effects remains important.

Earlier research has demonstrated the therapeutic potential of myricitrin, a flavonoid found in numerous fruits and vegetables, against dyslipidemia, NAFLD, and CVD [7,8,9,10]. Myricitrin (100 µM) treatment via oral gavage over a continuous 45-day period protected against dyslipidemia and atherosclerosis caused by a high-cholesterol diet (HCD) in rats [7]. Another study orally administered myricitrin (300 mg/kg/day) to streptozotocin (STZ)-treated mice for 8 weeks, and demonstrated its beneficial effects on diabetic cardiomyopathy [8]. Moreover, the oral administration of myricitrin (0.005%, *w*/*w*) for 5 weeks alleviated hepatic steatosis and inflammation in a diabetic mouse model subjected to a high-fat diet (HFD) and STZ treatment, and myricitrin treatment improved lipid accumulation in mouse hepatocytes exposed to ethanol [9,10]. However, the effects of myricitrin on HCD-caused hypercholesterolemia, NAFLD, and CVD in mouse models remain underexplored, particularly in the context of long-term dietary intervention. Among various animal models, the C57BL/6J mouse is widely recognized for its susceptibility to hyperlipidemia and atherosclerosis in response to HCD, establishing it as a recognized model for lipid metabolism and CVD research [11]. Additionally, its ability to develop hepatic steatosis, inflammation, and oxidative stress under HCD conditions makes it particularly valuable for investigating the progression of NAFLD and its links to cardiovascular risk factors [4,12,13,14,15]. Numerous studies have used the HCD-fed C57BL/6J mouse model to investigate the effects of different compounds on hyperlipidemia, NAFLD, and CVD [15,16,17,18].

Therefore, this study investigated the long-term (20 weeks) effects of dietary myricitrin (approximately 20 mg/kg body weight) in an HCD-fed C57BL/6J mouse model, focusing on its effects on plasma and hepatic lipids, plasma risk factors for CVD, hepatic lipid metabolism, and oxidative stress. By exploring these mechanisms, this study aimed to provide deeper insight into the feasibility of using myricitrin as a means for managing dyslipidemia and preventing NAFLD, ultimately contributing to a reduction in CVD risk.

## 2. Materials and Methods

### 2.1. Feeding Plan

Twenty-four male C57BL/6J mice (age of four weeks) acquired from Jackson Laboratories (Bar Harbor, ME, USA) were given a chow diet to acclimate. Following the adjustment period, the mice were randomized to receive either an HCD or an HCD supplemented with myricitrin (0.02%, *w*/*w*, purity of ≥99.0%, Sigma–Aldrich, St. Louis, MO, USA) (*n* = 12 for each group). The HCD diet used in this experiment was a commercial diet (D12336; Research Diets, New Brunswick, NJ, USA) containing 35 kcal% fat, 1.25% cholesterol, and 0.5% cholic acid. Each mouse was kept in a separate cage within an environment with controlled temperature (24 °C) and lighting (12-h light–dark cycle). They had unrestricted access to an experimental diet and water for 20 weeks. Daily food intake and weekly body weight were monitored. Mice exhibiting severe weight loss or signs of illness were designated as exclusion criteria, but no animals met these conditions during the study. Additionally, no data points were excluded during the analysis. Ethical approval for the research was secured from Kyungpook National University’s Ethics Committee (approval no. 2014-45).

### 2.2. Sample Collection

At the conclusion of the research, mice were kept without food for 12 h and treated with isoflurane (Baxter, Deerfield, IL, USA) to induce anesthesia prior to blood collection. After collecting heparin-treated plasma, livers were excised, weighed, and briefly stored in liquid nitrogen before being maintained at −70 °C.

### 2.3. Plasma Biomarkers

Plasma lipid concentrations were quantified with commercial kits for total cholesterol (TC; Asan, Seoul, Republic of Korea), triglycerides (Asan), high-density lipoprotein cholesterol (HDL-C; Abcam, Cambridge, UK), and low-density lipoprotein + very low density lipoprotein cholesterol (LDL + VLDL-C; Abcam, Cambridge, UK). Plasma levels of oxidized LDL (oxLDL; MyBioSource, San Diego, CA, USA), lipoprotein (a) (Lp(a); Elabscience, Wuhan, China), C-reactive protein (CRP; R&D Systems, Minneapolis, MN, USA), and plasminogen activator inhibitor-1 (PAI-1; Bio-Rad, Hercules, CA, USA) were quantified with assay kits. Plasma paraoxonase activity was assessed using the procedure outlined by Mackness et al. [19].

### 2.4. Hepatic Lipids

Hepatic lipid extraction was performed as previously described [20]. Subsequently, ethanol was added to the resulting dried lipid substances. After emulsifying, the same enzymatic kits applied to plasma analyses were utilized to measure hepatic triglyceride and cholesterol levels.

### 2.5. Histology

Hepatic tissues from all animals were preserved in fixatives and paraffinized, and then sectioned for histological staining. Hepatic lipid droplet and collagen deposition were examined employing a Nikon light microscope (Tokyo, Japan) at 200× magnification.

### 2.6. Lipid Peroxidation

Thiobarbituric acid reactive substances (TBARS) levels in the erythrocytes and liver, which represent a valuable indicator of lipid peroxidation, were quantified following the procedure outlined by Ohkawa et al. [21].

### 2.7. Enzyme Activity

The 3-hydroxy-3-methylglutaryl-CoA reductase (HMGCR) and acyl-CoA:cholesterol acyltransferase (ACAT) activities in the hepatic microsomes were quantified in accordance with earlier methods [22,23]. The superoxide dismutase (SOD), catalase, and glutathione peroxidase (GPX) activities were quantified using erythrocytes and the cytosol or mitochondria of the liver tissue. To assess SOD activity in erythrocytes and hepatic cytosol, spectrophotometric measurements were performed based on pyrogallol autoxidation inhibition [24]. Catalase activity in erythrocytic and hepatic mitochondria was assessed following previously established protocols [25], wherein the reduction in hydrogen peroxide was observed using a spectrophotometer. GPX activity in the erythrocytes and hepatic cytosol was measured using a spectrophotometric assay following previously described procedures [26]. Enzyme activities were normalized to the protein amount quantified [27] or the hemoglobin concentration estimated using a commercial assay kit (Sigma-Aldrich, St. Louis, MO, USA).

### 2.8. Gene Expression

To assess the effects of myricitrin on cholesterol metabolism in HCD-fed mice, the mRNA expression of genes involved in cholesterol synthesis, esterification, efflux, and clearance (HMGCR, ACAT, SREBP2, ABCA1, and LDLR) were analyzed. Total RNA isolation from liver was conducted using the TRIzol™ Reagent (Invitrogen Life Technologies, Grand Island, NE, USA), followed by cDNA synthesis employing QuantiTect^®^ reverse transcription kit (Qiagen, Hilden, Germany). A CFX96TM real-time system (Bio-Rad) was used to detect changes in gene expression. The relative gene expression levels normalized by GAPDH were quantified using the 2^−ΔΔCT^ approach.

### 2.9. Statistical Methods

SPSS version 11 was used for all statistical analysis. The mean values are displayed as standard errors (S.E.). Group differences were evaluated using Student’s *t*-test, with *p*-values < 0.05 regarded as statistically significant, after performing Levene’s test to check variance homogeneity. Sample size was determined based on previous studies showing significant effects of the intervention. At least ten mice per group were needed to identify differences in lipid profiles, with a power of 0.80 and an α level of 0.05 (G Power version 3.1). However, to account for potential variability and ensure more robust statistical power, 12 mice per group were used in the current study.

## 3. Results

### 3.1. Food Intake, Body Weight, Plasma Lipids, and Risk Factors for CVD

**Figure 1:**
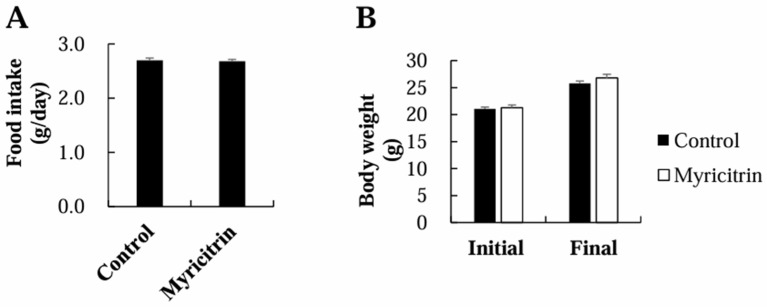
Impact of myricitrin on food consumption (**A**) and body weight (**B**) in mice given an HCD. Each column represents mean ± S.E. (*n* = 12). HCD, high-cholesterol diet.

**Figure 2:**
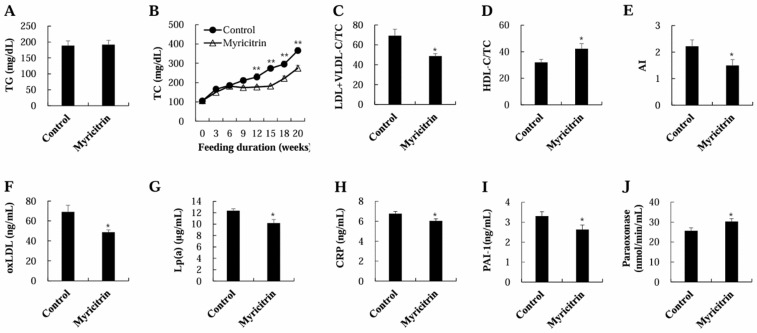
Impact of myricitrin on levels of plasma TG (**A**), TC (**B**), proportions of LDL/VLDL-C and HDL-C/TC (**C**,**D**), atherogenic index (**E**), oxLDL (**F**), Lp(a) (**G**), CRP (**H**), PAI-1 (**I**), and paraoxonase (**J**) in mice given an HCD. The results are mean ± S.E. (*n* = 12). * *p* < 0.05 and ** *p* < 0.01.

Food consumption or body weight remained unaltered (Figure 1). Plasma triglyceride concentrations were similar between the two groups (Figure 2A). However, at 12, 15, 18, and 20 weeks of myricitrin supplementation, the plasma TC concentrations were meaningfully decreased compared to the control group (Figure 2B). Moreover, myricitrin-supplemented mice exhibited a markedly lower plasma LDL + VLDL-C/TC proportion and a significantly higher HDL-C/TC proportion than control mice, resulting in a marked reduction in atherogenic index (AI) in the myricitrin group (Figure 2C–E). Myricitrin supplementation also led to a notable reduction in the plasma levels of risk factors for CVD, including oxLDL, Lp(a), CRP, and PAI-1, in HCD-fed mice, whereas the cardioprotective paraoxonase activity was markedly increased in the plasma of myricitrin-supplemented mice (Figure 2F–J).

### 3.2. Liver Weight, Lipid Content, Histology, and Cholesterol Metabolism Regulation

**Figure 3:**
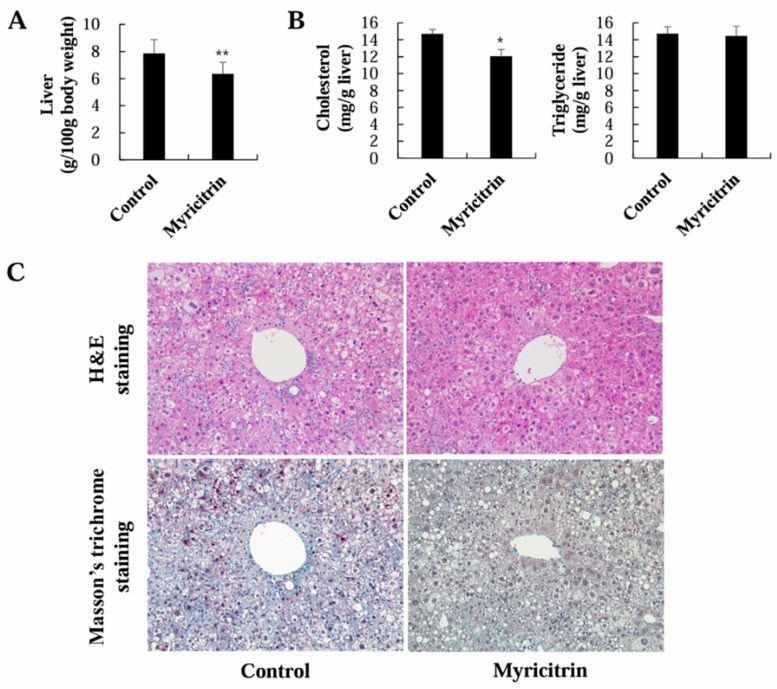
Impact of myricitrin on liver weight (**A**), lipid content (**B**), and histology (**C**) in mice given an HCD. (**A**,**B**) Each column represents mean ± S.E. (*n* = 12). * *p* < 0.05 and ** *p* < 0.01. (**C**) Microscopic image of liver at 200× magnification.

Myricitrin significantly decreased the liver weight of HCD-fed mice (Figure 3A). Hepatic cholesterol content was also meaningfully lowered by the myricitrin supplementation, although myricitrin did not change the hepatic triglyceride content in HCD-fed mice (Figure 3B). Histological analysis revealed pronounced morphological alterations and fibrosis in the livers of mice given an HCD, characterized by disorganized hepatocyte arrangement, lipid droplet accumulation, and collagen deposition. In contrast, myricitrin slightly improved the morphological alterations seen in H&E staining and marginally reduced the collagen deposition seen in Masson’s trichrome staining, indicating its protective effect against HCD-induced liver damage (Figure 3C).

**Figure 4:**
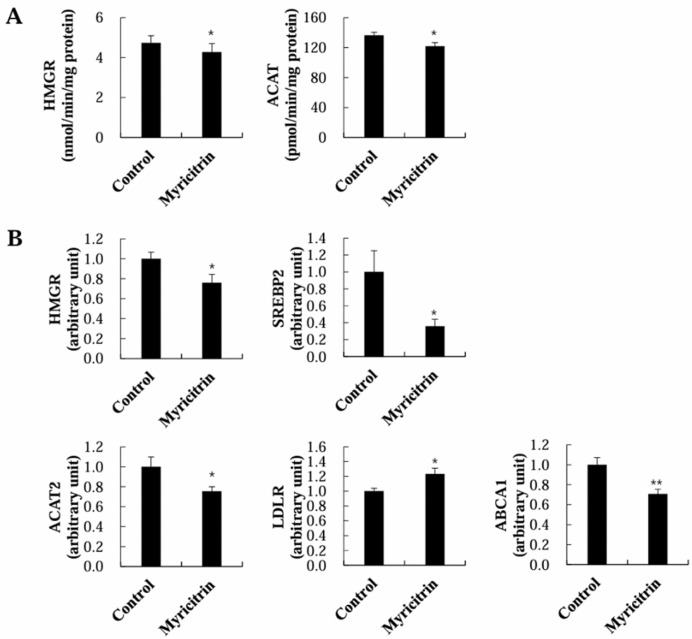
Impact of myricitrin on cholesterol metabolism-related enzyme activity (**A**) and gene mRNA expression (**B**) in livers of mice given an HCD. Each column represents mean ± S.E. (*n* = 12). * *p* < 0.05 and ** *p* < 0.01.

To investigate the mechanisms underlying the reduction in plasma and hepatic cholesterol levels by myricitrin in HCD-fed mice, the activities of enzymes and the mRNA expression of genes concerned with cholesterol synthesis and metabolism in the liver were analyzed. Myricitrin-treated mice exhibited meaningful decreases in the activities of hepatic HMGCR and ACAT, enzymes responsible for cholesterol synthesis and esterification, respectively (Figure 4A). These genes’ mRNA expressions were similarly affected by myricitrin (Figure 4B). Additionally, myricitrin supplementation significantly downregulated the mRNA expression of SREBP2 (an important transcription factor that regulates cholesterol synthesis) and ABCA1 (a gene involved in cholesterol efflux from the liver) in the liver (Figure 4B). Conversely, myricitrin supplementation upregulated the mRNA expression of LDLR, an essential receptor for LDL cholesterol clearance from the bloodstream, in the liver (Figure 4B).

### 3.3. Antioxidant Defenses in the Liver and Erythrocytes

**Figure 5:**
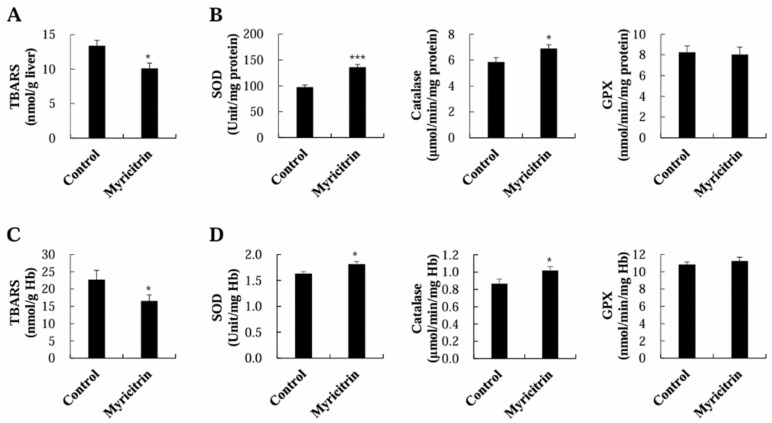
Impact of myricitrin on TBARS levels (**A**,**C**) and antioxidant enzymes activities (**B**,**D**) in erythrocytes and livers of mice given an HCD. Each column represents mean ± S.E. (*n* = 12). * *p* < 0.05 and *** *p* < 0.001.

To understand the effects of myricitrin on antioxidant defense in HCD-fed mice, TBARS and antioxidant enzyme activity in the liver and erythrocytes were analyzed. As anticipated, myricitrin markedly lowered the TBARS levels in both the liver and erythrocytes of HCD-fed mice (Figure 5A). The activation of SOD and catalase in both the liver and erythrocytes was observed in myricitrin-supplemented mice (Figure 5B). Yet, no meaningful differences were found in hepatic and erythrocytic GPX activities between the two groups (Figure 5B).

## 4. Discussion

This research examined the impact of myricitrin on plasma lipid profiles, CVD risk markers, hepatic lipid metabolism, and antioxidant defenses in HCD-fed mice. Our findings have demonstrated that myricitrin meaningfully reduced plasma and hepatic cholesterol levels and alleviated HCD-induced hepatic lipid droplet and collagen accumulation. In addition, myricitrin decreased key CVD risk markers, including plasma oxLDL, Lp(a), CRP, and PAI-1, although arterial improvement was not directly assessed. It also enhanced antioxidant defenses by increasing plasma paraoxonase and activating SOD and catalase in erythrocytes and the liver, accompanied by reductions in TBARS levels. These results suggest that dietary myricitrin may be helpful for treating HCD-induced hypercholesterolemia and NAFLD, with potential benefits for reducing CVD risk.

A key finding of this study was the effect of myricitrin on plasma lipid profiles and CVD risk factors. Despite no noticeable alterations in food consumption, body weight, or plasma triglyceride levels, dietary myricitrin supplementation led to a substantial reduction in plasma TC levels over several weeks. This is consistent with a prior study reporting the cholesterol-lowering effects of myricitrin treatment via oral gavage over a short-term period of 45 days in rats fed an HCD [7]. In that study, myricitrin treatment reversed HCD-caused increases in serum TC and LDL concentrations, while preventing a decrease in serum HDL levels [7]. Similarly, the present study showed that the long-term administration of dietary myricitrin improved plasma lipid ratios by decreasing the LDL + VLDL-C/TC ratio and increasing the HDL-C/TC ratio. These changes resulted in a notable reduction in the AI, which is a key marker for predicting CVD risk.

The cardioprotective effect of myricitrin was supported by the decreased plasma levels of several CVD risk factors, including oxLDL, Lp(a), CRP, and PAI-1. Notably, oxLDL functions as a key driver in atherosclerosis pathogenesis [28,29]. Higher plasma oxLDL concentrations have been observed in subjects with atherosclerotic plaques [30], and are indicative of future cardiovascular risk prediction in healthy populations [31]. Similarly, Lp(a), a lipoprotein resembling LDL, but containing apolipoprotein(a), contributes to the CVD progression by promoting both atherosclerosis and thrombosis [32]. The independent contributions of elevated Lp(a) levels in cardiovascular events highlight importance of this in guiding primary prevention strategies for CVD [33]. CRP, a protein that responds to inflammation, is another significant predictive marker of cardiovascular events [34]. Chronically elevated CRP levels indicate systemic inflammation, which is a major contributor to CVD [34]. Elevated CRP levels are linked to higher risks of heart attack, stroke, and other cardiovascular events, either independently or with others [34]. Finally, elevated levels of PAI-1, a protein that inhibits fibrinolysis (the process responsible for preventing blood clots), have been linked to impaired clot resolution, thereby increasing the risk of thrombotic events [35]. Therefore, the observed decreases in these markers reflect the ability of myricitrin to lower CVD risk.

In addition, myricitrin markedly activated plasma paraoxonase, which plays a crucial role in preventing oxLDL formation [36]. Enhanced paraoxonase activity is associated with decreased oxLDL levels and is negatively correlated with the severity and extent of atherosclerotic CVD [37,38]. Paraoxonase is an HDL-linked enzyme with antioxidant properties. It is thought to inhibit oxLDL production through multiple mechanisms, including the enhancement of HDL functionality and the hydrolysis of oxidized phospholipids [39,40].

Along with the activation of plasma paraoxonase, myricitrin promoted the activation of other antioxidant enzymes (e.g., SOD, catalase) in erythrocytes. Erythrocytes are susceptible to reactive oxygen species attack due to their important action as oxygen-carriers. Therefore, they function as an essential part of the circulating enzymatic defense system against oxidative stress [41]. An increase in the activities of erythrocyte SOD and catalase could be associated with a decrease in plasma oxLDL formation because oxLDL is formed when LDL particles are exposed to oxidative stress [42,43]. Oxidative stress reduction by antioxidant enzymes may help protect lipids from peroxidation and reduce oxLDL formation in the plasma. Decreased oxidative stress is reflected by decreased levels of erythrocyte TBARS, lipid peroxidation marker, and plasma oxLDL. These results suggest an association between antioxidant enzyme activation and cardioprotective effects. Although not specifically investigated in this study, it is possible that the balance between reactive oxygen species and reactive nitrogen species also plays a critical role in oxidative stress-related damage [44]. Additionally, non-enzymatic antioxidants (e.g., glutathione, vitamin E) may contribute to the overall antioxidant defense system, potentially complementing the enzymatic activities of SOD and catalase [45].

A high cholesterol intake is linked to an elevated risk of NAFLD as well as CVD [46]. Although NAFLD is classically characterized by increased hepatic triglycerides levels, cholesterol overload is also observed in NAFLD [47]. Disruptions in cholesterol production and metabolism in the liver can contribute to cholesterol accumulation in NAFLD, leading to liver injury [47,48]. Increased expressions of hepatic HMGCR, an enzyme that limits cholesterol production, and SREBP2, a principal activator of HMGCR [49], were observed in NAFLD, whereas the expression of hepatic LDLR was decreased [48]. LDLR facilitates the uptake of blood LDL-C into the liver [50], and the increased influx suppresses HMGCR by inhibiting SREBP 2 activation [51].

In this research, myricitrin significantly lowered the liver weight and hepatic cholesterol content in mice given an HCD. The lower cholesterol levels in the plasma and liver might result from the promotion of plasma cholesterol transport to the liver, thereby inhibiting hepatic cholesterol biosynthesis through decreased hepatic SREBP2 and HMGCR mRNA expression or activity, along with the increased hepatic LDLR mRNA expression. Additionally, the inhibition of hepatic ACAT mRNA expression and activity by myricitrin supplementation can contribute to the reduction in hepatic cholesterol levels because ACAT is responsible for converting free cholesterol into cholesterol esters for storage. As cholesterol is removed from the blood and not produced or stored readily within the liver, this helps maintain lower cholesterol concentrations in both the plasma and liver. Moreover, the downregulation of hepatic ABCA1, which facilitates hepatic cholesterol export [52], may have contributed to lower plasma cholesterol levels by limiting cholesterol export from the liver to the blood. In atherogenic diet-fed LDLR-knockout mice, hepatic ABCA1 deletion significantly decreased plasma VLDL and LDL levels, and showed a protective effect against atherosclerosis [53].

Our histological analysis also showed that myricitrin treatment led to minor improvements in disorganized hepatocyte arrangement, lipid droplet accumulation, and collagen deposition. These findings suggest that myricitrin may offer moderate protective effects against HCD-induced liver damage. Dietary cholesterol promotes hepatic injury by increasing the oxidative stress [54]. Oxidative stress contributes significantly to NAFLD pathogenesis [55]. The disruption of antioxidant defense systems, such as antioxidant enzymes, can lead to oxidative stress associated with NAFLD [56,57,58]. SOD deficiency in mice leads to lipid and collagen accumulation in the liver [46,47], and catalase-knockout mice show exacerbated HFD-induced liver injury, including hepatic lipid accumulation [58]. Interestingly, myricitrin activated SOD and catalase and decreased TBARS concentrations in the liver, suggesting that it could help mitigate oxidative liver damage and aid in alleviating NAFLD.

## 5. Conclusions

In conclusion, myricitrin has multifaceted beneficial effects on lipid metabolism, CVD risk factors, liver health, and oxidative stress in HCD-fed mice. These findings suggest that myricitrin has the potential to be a valuable therapeutic agent for managing hypercholesterolemia and reducing the associated cardiovascular and liver diseases. Further studies, including clinical trials, are warranted to confirm these effects in humans and explore the underlying mechanisms in more detail.

## Data Availability

The original contributions and data presented in this study are included in the article. Further inquiries can be directed to the corresponding author when necessary. The data are not publicly available due to the ethical guidelines for animal research.
